# Inherited Syphilis

**Published:** 1908-04

**Authors:** 


### INHERITED SYPHILIS.

The question of syphilis being inherited alone from the father, the mother remaining uninfected, has always been an interesting, though obscure one, and many elaborate arguments have been advanced to prove and disprove this theory. A new field of investigation has been opened by the discovery of the sperochaeta pallida, and its almost universal acceptance as the cause of syphilis, or at least an etiological factor. Lucas* has recently asserted that purely fraternal syphilis being transmitted to offspring is a view no longer tenable. He claims that it seems impossible that a motile organism exceeding in length the diameter of an ovum, could possibly penetrate and multiply in it without destroying it. Therefore, he lays it down as an axiom that inheritance is invariably through the syphilized mother, which conclusion is supported by Colles' law. When virulent, the spirochetes penetrate the charion or placenta and occasion miscarriages, macerated fetuses or premature births: when the virus is attenuated (rather when the host's resistance is greater) the placenta protects the developing fetus, and infection takes place only through the umbilical vein on the separation of the placenta, thus explaining the appearance of the secondary symptoms in the infant from two to three months after birth. In these cases the separation of the placenta is the first stage, and corresponds to the chancre of acquired syphilis.

Kassowitz, in an able paper in 1876, opened the study of this subject, and his arguments in favor of paternal heredity have not been carefully answered from the opposite view until a recent publication by Matzenauer, who, with Lucas and Sturgis, insist that every syphilitic child has a syphilitic mother.

In this connection I have consulted Keyes Monograph on Syphilis to obtain his views of the subject. He says there are five ways in which a fetus or child may become infected with syphilis: 1. at the moment of conception, by the father's semen; 2. at the moment of conception by the mother's ovum; 3, by means of the placental circulation to the healthy fetus; 4, infection during parturition; 5, after birth by a kiss, or in suckling.

Although admitting that almost universally syphilographers believe in infection through the semen—the mother escaping infection—pathologists have always maintained a reasonable doubt concerning this unique seminal transmission, which is not even alleged for any disease except syphilis. Keyes* arrays the evidence upon each side. In favor of paternal heredity is alleged the obvious fact that the father and child are certainly syphilitic, and the mother apparently sound.

Against it is urged that the mother is not absolutely sound; and this is exhibited in various ways. In the first place, she is subject to Colles' law—the child being able to infect any non-syphilitic person except its own mother. This immunity to syphilis exhibited by the mother of a syphilitic child means that she is in some sense syphilitic. But in what sense? Either she has absorbed from the fetus or developed some immunizing substance, or, on the other hand she is actually syphilitic. In favor of the former theory are: First, certain reported exceptions to Colles' law, the mother developing chancre of the nipple and a general syphilis after nursing her infant. (Matzenauer rejects all these exceptions as being unreliable.)

Second, the repeated insistance of innumerable observers that in such instances further syphilitic pregnances may be prevented by *anti-syphilitic treatment of the father alone.*

Against it are:

1. Our inability to evoke a similar experimental immunity in man or monkey.

2. The fact that some ninety per cent. of syphilitic women do not have, or do not remember having, any early symptoms of the disease.

3. The frequency of the appearance in later years of tertiary lesions upon these apparently clean mothers. In such instance we may fairly say that the mother was syphilitic, but skipped her early symptoms.

4. The decreasing intensity of the infection in successive pregnancies; the one thing most likely to affect the vitality of the fetus and its susceptibility to disease is the condition of the placenta, and Matzenauer has attempted to show that the decreasing virulence of children goes hand in hand with decreasing syphilitic inflammation of the placenta.

Briefly stated, the above are a few of the facts as elucidated by men who have given the subject much thought and study. That paternal heredity ever occurs, Keyes says he does not

*Lancet London, Feb. 1st '08

know, but claims that the frequency of the appearance of tertiary lesions in apparently healthy mothers surely proves that it is less common than is generally believed.

